# The Hospital Articles on Blackwell’s Island and the New York Hospital

**Published:** 1860-01

**Authors:** 


*The Hospital Articles on Blackwell's Island and the New York Hospital.*—We omitted to state that the article on Blackwell's Island in our last number, was furnished by Dr. SANGER. The article on the New York Hospital, which we have in this issue, was furnished by Dr. HENRY N. FISHER, Resident Surgeon.

